# A seven-gene cluster in *Ruminiclostridium cellulolyticum* is essential for signalization, uptake and catabolism of the degradation products of cellulose hydrolysis

**DOI:** 10.1186/s13068-017-0933-7

**Published:** 2017-10-30

**Authors:** Aurélie Fosses, Maria Maté, Nathalie Franche, Nian Liu, Yann Denis, Romain Borne, Pascale de Philip, Henri-Pierre Fierobe, Stéphanie Perret

**Affiliations:** 1 Aix Marseille Univ, CNRS, LCB, Marseille, France; 2 Aix Marseille Univ, CNRS, AFMB, Marseille, France; 3 Aix Marseille Univ, CNRS, Plateforme Transcriptome, Marseille, France

**Keywords:** ABC-transporter, Three-component system, Cellobiose, Cellodextrins, Cellobiose phosphorylase

## Abstract

**Background:**

Like a number of anaerobic and cellulolytic Gram-positive bacteria, the model microorganism *Ruminiclostridium cellulolyticum* produces extracellular multi-enzymatic complexes called cellulosomes, which efficiently degrade the crystalline cellulose. Action of the complexes on cellulose releases cellobiose and longer cellodextrins but to date, little is known about the transport and utilization of the produced cellodextrins in the bacterium. A better understanding of the uptake systems and fermentation of sugars derived from cellulose could have a major impact in the field of biofuels production.

**Results:**

We characterized a putative ABC transporter devoted to cellodextrins uptake, and a cellobiose phosphorylase (CbpA) in *R. cellulolyticum*. The genes encoding the components of the ABC transporter (a binding protein CuaA and two integral membrane proteins) and CbpA are expressed as a polycistronic transcriptional unit induced in the presence of cellobiose. Upstream, another polycistronic transcriptional unit encodes a two-component system (sensor and regulator), and a second binding protein CuaD, and is constitutively expressed. The products might form a three-component system inducing the expression of *cuaABC* and *cbpA* since we showed that CuaR is able to recognize the region upstream of *cuaA*. Biochemical analysis showed that CbpA is a strict cellobiose phosphorylase inactive on longer cellodextrins; CuaA binds to all cellodextrins (G2–G5) tested, whereas CuaD is specific to cellobiose and presents a higher affinity to this sugar. This results are in agreement with their function in transport and signalization, respectively. Characterization of a *cuaD* mutant, and its derivatives, indicated that the ABC transporter and CbpA are essential for growth on cellobiose and cellulose.

**Conclusions:**

For the first time in a Gram-positive strain, we identified a three-component system and a conjugated ABC transporter/cellobiose phosphorylase system which was shown to be essential for the growth of the model cellulolytic bacterium *R. cellulolyticum* on cellobiose and cellulose. This efficient and energy-saving system of transport and phosphorolysis appears to be the major cellobiose utilization pathway in *R. cellulolyticum*, and seems well adapted to cellulolytic life-style strain. It represents a new way to enable engineered strains to utilize cellodextrins for the production of biofuels or chemicals of interest from cellulose.

**Electronic supplementary material:**

The online version of this article (doi:10.1186/s13068-017-0933-7) contains supplementary material, which is available to authorized users.

## Background

Cellulose is the most abundant polysaccharide on earth, mostly found in plant-cell wall. This polymer is made up of linear chains of glucosyl residues linked through β-1,4 glycosidic bonds, and arranged in a quasi-crystalline structure that makes it highly recalcitrant to enzymatic hydrolysis. This large reservoir of glucose represents a remarkable potential renewable source of energy to produce biofuels or chemicals as an alternative to fossil-derived products. Conversion of cellulose into biofuel through fermentation process depends on its efficient enzymatic breakdown into fermentable sugars, but also relies on the subsequent uptake and fermentation of the degradation products by fermentative microorganisms.

The model bacterium *Ruminiclostridium cellulolyticum* is an anaerobic organism able to grow on cellulose as a unique carbon source, as well as on soluble sugars such as xyloglucan, cellobiose and to a lesser extent glucose [1–3]. The bacterium performs efficient extracellular degradation of plant cell wall polysaccharides into fermentable sugars thanks to multienzymatic complexes called cellulosomes [4, 5]. This enzymatic system has been extensively studied over the past 20 years, mining the enzymatic diversity, and understanding the cellulosomes’ assembly and functioning in plant cell wall degradation process [4–9]. However uptake of the released cellodextrins is crucial for growth on cellulose, and little is known about their transport and utilization into *R. cellulolyticum*, as well as in other cellulosomes-producing bacteria.

In microbes, sugar uptake is usually mediated by phosphotransferase system (PTS), secondary transporters energized by proton-motive force, or ABC transporter functioning with ATP hydrolysis. In the genome of *R. cellulolyticum*, no PTS system-encoding genes were found, whereas ABC transporters seem to be largely involved in the uptake of saccharides by this bacterium [10]. ABC-type importers are composed of two transmembrane domains forming the channel of the transporter, two intracellular nucleotide-binding domains (NBD) energizing the system through ATP hydrolysis, and an additional extracellular solute binding protein (SBP) essential to capture and deliver the substrate to the cognate translocator. In *R. cellulolyticum*, only one ABC transporter was studied to date. It was shown to be specific of 4-glucosyl-backbone xyloglucan dextrins which are subsequently degraded in the cytoplasm by three specific enzymes [3]. In some other Gram-positive cellulolytic bacteria and archaea, some binding proteins of ABC transporters were shown to bind to cellobiose and/or cellodextrins; the role of the cognate ABC-transporters during the growth on cellulose was not established [11–15].

In the cellulolytic bacteria, imported cellodextrins are subsequently degraded by the action of intracellular cellodextrin phosphorylases or cellobiose phosphorylases which belong to the Family-94 of glycosyl hydrolases [16–19]. These enzymes convert cellodextrins of n glucosyl residues in glucose-1-P and a cellodextrin of n-1 glucosyl residues without investment of ATP. They are therefore assumed to save one ATP molecule when the phosphorylated monosaccharide enters the glycolytic pathway. This energy-saving pathway is of particular importance in anaerobic bacteria in which the yield of ATP is usually low.

Genome analysis of *R. cellulolyticum* reveals that four genes encode putative phosphorylases located at loci Ccel_1439, 2109, 2354, 3412 [4]. Among them, only the gene located at the locus Ccel_2109 is adjacent to genes encoding a putative ABC transporter and a three-component system forming a cluster of genes. This cluster was named *cua* for cellulose utilization associated [10]. In the present report, we focused on this gene cluster, and explored its role in cellobiose and cellulose degradation products signalization, uptake, and catabolism.

## Results

### Sequence analysis of *cua* gene cluster and its products

**Fig. 1:**
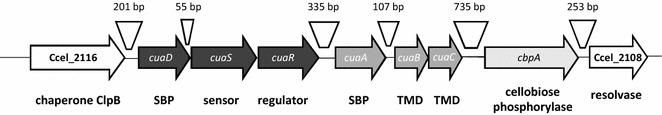
The *cua* gene cluster and its neighboring genes. The genes encoding the putative three-component system are represented in black (*cuaDSR*), the ABC transporter in dark gray (*cuaABC*), and the putative cellobiose phosphorylase in gray (*cbpA*). Sizes (in bp) of intergenic sequences are indicated above. The surrounding genes are shown in white. Putative function of the gene products is indicated below, with SBP for solute binding protein, TMD for transmembrane domain

The *cua* gene-cluster from loci Ccel_2109 to Ccel_2115 encompasses seven genes predicted to encode (i) a solute binding protein (CuaD), (ii) a putative two-component system composed of a sensor (CuaS) and a response regulator (CuaR), (iii) a solute binding protein CuaA and two integral membrane proteins CuaB and C, forming a putative ABC transporter, and (iv) a putative GH94 cellobiose phosphorylase named CbpA in the present study (Fig. 1).

Upstream and downstream of the cluster, the genes at loci Ccel_2116 and Ccel_2108 encode a putative chaperon protein and a resolvase, respectively. In the cluster, overlapping genes or short intergenic region are observed between the genes *cuaD*, *cuaR*, and *cuaS*, and between *cuaA*, *cuaB*, and *cuaC*. The largest intergenic region (735 bp) is located between the genes *cuaC* and *cbpA* (Fig. 1).

The putative sensor CuaS exhibits features characteristic of bacterial histidine kinase proteins with two predicted transmembrane helices, delimiting a 268 amino acids external loop and a conserved C-terminal cytoplasmic region containing an ATP-binding kinase domain. The putative response regulator CuaR belongs to the AraC/XylS family. Both CuaA and CuaD are predicted to be ABC transporters solute binding proteins (SBP) belonging to Family-1, but they share only 14% sequence identity. This family includes proteins displaying affinity to oligosaccharides, whereas the Family-2 SBPs target monosaccharides [20]. As for SBPs found in other Gram-positive bacteria, an N-terminus typical lipoprotein signal peptidase II is predicted for both proteins. The putative cleavage site is located at amino acids 22 and 20 for CuaA and CuaD, respectively, and the mature proteins are predicted to bear an N-terminal acylated cysteine. CuaB and C are putative transmembrane domains, each of them containing six transmembrane-spanning helices, and the typical signal sequence found in integral membrane proteins of ABC transporters [20, 21].

### Analysis of the substrates of CbpA, CuaD, and CuaA

As the *cua* cluster seems to be implicated in cellobiose/cellulose metabolism [10], we first investigated the binding abilities of the SBPs and characterized the activity of the putative phosphorylase towards cellodextrins. Three recombinant proteins, rCbpA, rCuaA, and rCuaD, were overproduced in *E. coli* and purified. Activity and specificity of rCbpA was analyzed on cellobiose and longer cellodextrins by quantifying both the released products including α-d-glucose-1-phosphate, and the consumption of the substrate. The enzyme displays phosphorolytic activity on cellobiose (G2) specifically, with *K*_M_ and *k*_cat_ values of 2.85 ± 0.47 mM and 1458.4 ± 108 min^−1^, respectively, but no activity was detected on longer cellodextrins (G3–G5).

The ability of rCuaD and rCuaA to bind to cellodextrins was analyzed using IsoThermal microCalorimetry (ITC). Both SBPs failed to bind to glucose and arabinose. rCuaA was shown to bind to all tested cellodextrins (G2–G5) (Table 1). In all cases, the titration curve fits with a single binding model with a calculated *n* (stoichiometry) of 1 (see Additional file 1). Analysis of the *K*_D_ values for the various cellodextrins in the case of CuaA indicates that the affinity is inversely proportional to the size of the dextrins (G2 > G3 > G4 > G5). On the other hand, rCuaD interacts only with cellobiose but with an affinity 10 times higher for the disaccharide than rCuaA. Although comparable Δ*G* values were obtained for both proteins (approx. − 9 kcal mol^−1^), the binding of rCuaA to cellobiose is characterized by a highly favorable enthalpy, whereas rCuaD interacts with cellobiose with both favorable enthalpy and entropy (Table 1), thereby indicating that rCuaA and rCuaD bind to cellobiose with different interaction modes. Overall, these results support the predicted role of these three proteins in the signalization, uptake, and degradation of cellobiose/cellodextrins.

**Table 1:** Binding of rCuaA and rCuaD to cellodextrins: thermodynamic parameters and dissociation constants

Protein	Substrate	*K*_*D*_ (nM)	Δ*G* (kcal mol^−1^)	Δ*H* (kcal mol^−1^)	− *T*Δ*S* (kcal mol^−1^)
rCuaD	G2	23 ± 3	− 9.95 ± 0.06	− 8.25 ± 0.18	− 1.70 ± 0.13
rCuaA	G2	240 ± 20	− 8.88 ± 0.04	− 33.30 ± 0.10	24.40 ± 0.14
	G3	405 ± 110	− 8.59 ± 0.16	− 31.90 ± 1.70	23.30 ± 1.54
	G4	644 ± 58	− 8.38 ± 0.65	− 15.14 ± 0.11	6.80 ± 0.03
	G5	1860 ± 130	− 7.69 ± 0.04	− 12.80 ± 0.08	5.12 ± 0.12

### Polycistronic organization and regulation of the expression of the *cua* genes

**Fig. 2:**
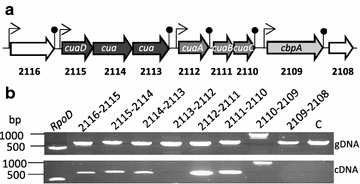
Transcriptional intergenic links in the *cua* cluster. **a** Genetic organization of *cua* cluster with localization of promoters (arrow) and terminators (stem-loop) predicted by BPROM and FindTerm softwares. **b** RT-PCR analysis of *cua* gene cluster. PCR amplification was performed on genomic DNA (upper lane) or cDNA (lower lane) obtained after reverse transcription from a cellobiose grown culture. The primers used to study the transcriptional links between two successive genes were localized at the end of the first gene for the direct primer and at the beginning of the second gene for the reverse primer. Genes are named using their locus-tag number except for the control *rpoD*. The negative control (C) corresponds to a PCR performed on cDNA using primers targeting an intergenic region between two divergent genes, for which amplification might be detected if genomic DNA is present

Based on their expression patterns determined by qPCR, two groups of genes were identified: the *cuaDSR* group which is constitutively expressed, and the group encompassing *cuaABC* and *cbpA* which is upregulated in the presence of cellobiose or cellulose (see Additional file 2) [10]. To further analyze if they form two distinct polycistronic units, transcriptional intergenic links between the *cua* genes and their adjacent genes were investigated. Using cDNA prepared from mRNA of a cellobiose grown culture, transcriptional links were observed between the successive genes Ccel_2116-*cuaD*-*cuaS*-*cuaR* and between the genes *cuaA*-*cuaB*-*cuaC*-*cbpA*, respectively (Fig. 2). In contrast, no amplification was detected between adjacent genes *cuaR* and *cuaA*, neither between the gene *cbpA* and the following gene located at locus Ccel_2108. These results support the presence of two transcriptional units on cellobiose, [*Ccel_2116*-*cuaD*-*cuaS*-*cuaR*] and [*cuaA*-*cuaB*-*cuaC*-*cbpA*]. Using bioinformatic tools, terminators were predicted downstream *cuaR,* and *cbpA* in agreement with the obtained results (Fig. 2). However, the presence of a promotor and a terminator was also predicted upstream and downstream of the gene at locus Ccel_2116, suggesting that this gene could also be expressed by the way of an independent transcriptional unit. However depending on its strength, the prediction of a terminator downstream of the gene at locus Ccel_2116 does not exclude the possibility of a transcriptional link with the following gene *cuaD* as it is experimentally observed. A terminator was also predicted at the end of *cuaA* although a transcriptional link was clearly detected with the following gene *cuaB*. This terminator might be involved in the regulation of the expression level of *cuaA* compared to *cuaB* and *C* that may lead to an excess of binding protein compared to the transmembrane channel as it was formerly observed for the maltose ABC transporter in *E. coli* [22].

Overall the results strongly suggest that the *cua* genes are organized in two transcriptional units: (*Ccel_2116*)-*cuaD*-*cuaS*-*cuaR* constitutively expressed, and *cuaA*-*cuaB*-*cuaC*-*cbpA* which is overexpressed in the presence of cellobiose and cellulose compared to arabinose.

### Role of the regulator CuaR

**Fig. 3:**
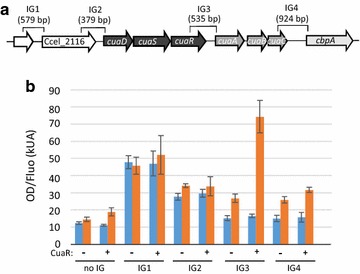
Targets of the response regulator CuaR. **a** Localization and size of the intergenic regions tested IG1, IG2, IG3, and IG4, in the *cua* cluster. **b** Fluorescence intensity of recombinant *E. coli* MG1655 containing pBAD24 or pBAD*cuaR* and different derivatives of the pUA66 carrying intergenic regions or not. Arabinose was added (red) or not (blue) to the medium. Arabinose allows induction of the expression of *cuaR* in the pBAD*cuaR* vector. Experiments were performed in triplicate and bars indicate the standard deviation

As the expression of some of the genes of the *cua* cluster is regulated, we tested the ability of CuaR to induce their expression by reconstitution of the induction pathway in *E. coli*. For that purpose, the gene *cuaR* was expressed in *E. coli* under the control of an arabinose inducible promoter in a pBAD24 vector (pBAD*cuaR*). The cells were co-transformed with low copy vector pUA66 derivatives. These vectors carry the *gfpmut2* reporter gene encoding a green fluorescent protein (GFP), transcriptionally fused to each of the intergenic sequences located upstream of the genes at loci Ccel_2116 (IG1), Ccel_2115 (*cuaD*, IG2), Ccel_2112 (*cuaA*, IG3) or Ccel_2109 (*cbpA* IG4). A pUA66 vector without any intergenic sequence was used as a negative control (Fig. 3). The fluorescence level measured directly in the cells thus reflects the expression level of the reporter gene. With pBAD24 vector, a basal level of fluorescence was measured for each intergenic region. The fact that this basal level was rather high in the case of the intergenic region IG1 suggests that this sequence was at least partly recognized by an *E. coli* resident regulator/polymerase. With pBAD*cuaR* and in the presence of arabinose, a drastic increase of the fluorescence was only observed when the reporter gene was fused to IG3 (Fig. 3). This result demonstrates that, in *E. coli*, CuaR is able to recognize the region upstream of *cuaA*, and induces the expression of the reporter gene. The gene *cuaR* therefore probably acts as an activator of the transcription of *cuaABC* and *cbpA* in *R. cellulolyticum*.

### Growth of a *cuaD* mutant strain on cellobiose

To investigate the role of these genes in *R. cellulolyticum*, the group II intron-based Clostron technology was used to inactivate the genes *cuaD* and *cuaA* [23]. The *cuaD* mutant strain was easily obtained and named MTL*cuaD*, whereas *cuaA* mutant could not be obtained in spite of using different targets within *cuaA*.

**Fig. 4:**
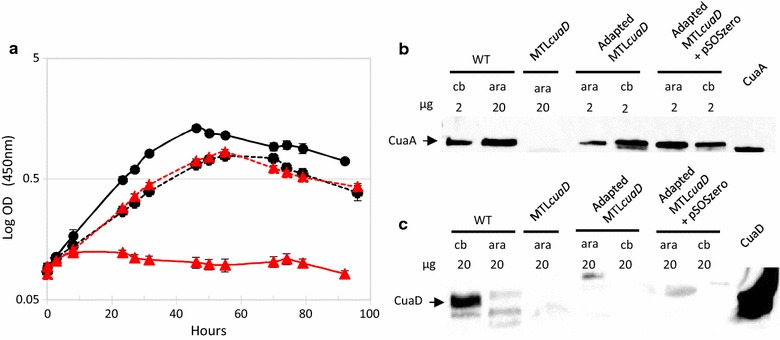
Characterization of *R. cellulolyticum* wild-type and MTL*cuaD* strains. **a** Growth of the wild-type (black) and the mutant (red) strains on minimal medium containing 2 g L^−1^ arabinose (dotted line) or cellobiose (solid line). Experiments were performed in triplicate and bars indicate the standard deviation. **b**, **c** Western blot analyses of the membrane fraction obtained for wild-type, MTL*cuaD* strains, and cellulose adapted MTL*cuaD* strains, using anti-CuaA (**b**) or anti-CuaD (**c**) antiserum. The amounts of membrane fraction loaded are given in µg and are obtained from arabinose (ara) or cellobiose (cb) grown cells

On arabinose, MTL*cuaD* and wild-type strains showed similar growth, but in contrast to the wild-type strain the mutant strain failed to grow on cellobiose, thereby indicating that *cuaD* is critical for cellobiose utilization (Fig. 4). To analyze further this mutant, the presence of CuaD and CuaA was assayed by western blot analyses using antisera raised against rCuaD and rCuaA, respectively. CuaD and CuaA were both detected in the membrane fraction of the wild-type strain grown on cellobiose or arabinose, but none of these binding proteins were detected in the mutant strain grown on arabinose (Fig. 4b, c). This observation strongly suggests that the inability of the MTL*cuaD* strain to grow on cellobiose is due to the fact that CuaA and most probably the other components of the ABC transporter are no longer synthesized. As we have shown (see above) that *cuaD, cuaS*, and *cuaR* are transcriptionally linked, the insertion of the type II intron in *cuaD* most probably induced a polar effect on the expression of the genes *cuaS* and *cuaR,* as already described for insertional type II intron inactivation in the *cip*-*cel* operon of *R. cellulolyticum* [24]. The expression level of the genes *cuaD*, *cuaS*, and *R* was shown to be about 10 time lower in the mutant strain compared to the wild-type strain both grown on arabinose, confirming the polar effect (see Additional file 3). The disability of the signalization system has severely hampered the expression of the genes *cuaABC*-*cbpA*, thus explaining the inability of the MTL*cuaD* to grow on cellobiose-based medium.

### Adaptation of *cuaD* mutant strain on cellulose

**Fig. 5:**
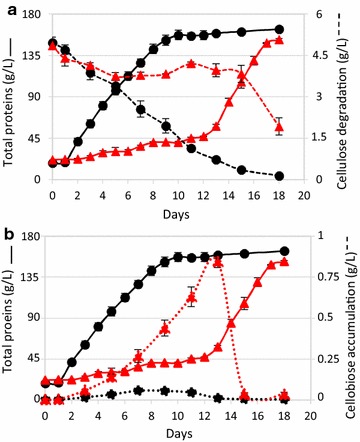
Growth of *R. cellulolyticum* wild-type and MTL*cuaD s*trains on cellulose. Wild-type (black) and MTL*cuaD* (red) strains were grown on minimal medium containing 5 g L^−1^ cellulose. **a** Cellulose consumption (dotted lines) and growth curve (solid lines). **b** Concentration of cellobiose in the culture supernatant (dotted lines) and growth curve (solid lines). Experiments were performed in triplicates and bars indicate standard deviation

**Fig. 6:**
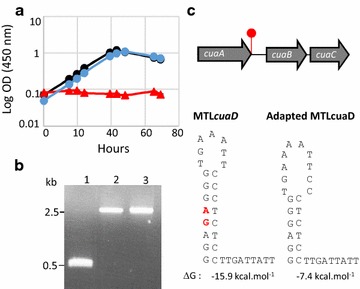
Characterization of the adapted mutant strain. **a** Growth of *R. cellulolyticum* wild-type (black), MTL*cuaD* (red), and adapted MTL*cuaD* mutant strain (blue) on minimal medium containing cellobiose (2 g L^−1^). **b** Molecular analysis of the type II intron insertion in *cuaD*. PCR was performed using the primers pair CuaD_734Dir/CuaD_1187Rev on cells from wild-type (1), MTL*cuaD* mutant (2) or adapted MTL*cuaD* mutant (3) strains. Migration at 2.5 kb indicates intron insertion. **c** A deletion of two bases (in red) is observed in the stem part of the terminator located downstream *cuaD* (top panel and below left panel), thus reducing the strength of the terminator in adapted MTL*cuaD* strain (below right panel). The strength of the terminator is calculated using ARNold on line software

To further analyze its phenotype, the MTL*cuaD* strain was grown on minimal medium containing crystalline cellulose and growth was followed by measuring the protein content (Fig. 5). The wild-type strain grew up to 150 µg mL^−1^ from day 1 to day 8, whereas the mutant strain grew very slowly up to day 11–12, after which a growth at the same rate as the wild-type strain was observed. As the enzymatic degradation of the cellulose by the cellulosomes during the culture generates an array of cellodextrins ranging from 2 to 6 glucosyl residues, we analyzed the soluble sugars in the culture supernatant. This analysis revealed an accumulation of cellobiose in the MTL*cuaD* culture during the first 12 days along with a slight consumption of cellulose (20%), whereas nearly no soluble sugars were detected in the supernatant of the wild-type culture (Fig. 5a, b). After 12 days, the cellobiose disappeared rapidly in the MTL*cuaD* culture, thereby indicating that the strain was suddenly able to import cellobiose. At the same time, the strain started to consume cellulose faster and to grow with a doubling time similar to the wild-type strain. This adapted mutant strain growing on cellulose was subsequently assayed on cellobiose-based medium and was found to grow as fast as the wild-type strain on the disaccharide (Fig. 6). The mutation introduced by the type II intron is still present in this adapted mutant strain (Fig. 6) and CuaD could not be detected, as in the original MTL*cuaD* strain (Fig. 4c). Nevertheless, western blot analyses revealed the presence of CuaA although this protein was undetectable in the original mutant strain (Fig. 4b). Altogether, these results strongly suggest that spontaneous mutation(s) occurred allowing the strain to adapt and to use the crystalline polysaccharide thanks to at least the presence of CuaA, suggesting that the growth on cellobiose and cellulose is strongly correlated to the presence of the Cua ABC transporter. The sequencing of the adapted MTL*cuaD* strain genome indicated that several mutations occurred (see Additional file 4). Among them, the most relevant mutation was a two-base pair deletion within the terminator stem between *cuaA* and *cuaB*, reducing the theoretical strength of the terminator by two (Fig. 6). In this context, it is hypothesized that this mutation may lead to a stabilization of the messenger transcribed from *cuaABC*-*cbpA*, thereby increasing the production of the Cua ABC transporter and CbpA, and thus the recovery of a normal growth on cellobiose and cellulose.

### Importance of the ABC transporter and CbpA for growth on cellobiose and cellulose

To further examine the role of the ABC transporter and of CbpA during the growth on cellobiose and cellulose-based media, we functionally complemented the MTL*cuaD* mutant strain with replicative vectors carrying either no expression cassette (pSOSzeroTm), the genes *cuaABC* (pSOS*cuaABC*), the genes *cuaABC*-*cbpA* (pSOS*cuaABC*-*cbpA*), or the gene *cbpA* alone (pSOS956*cbpA*). All the genes were cloned under the control of a constitutive promoter, but the native gene organization found in *R. cellulolyticum* was maintained except for the vector pSOS*cuaABC*-*cbpA* where the large intergenic region (735 bp) naturally occurring between *cuaC* and *cbpA* was shortened to 30 bp, including a ribosome binding site to secure the expression of *cbpA* into the operonic structure.

**Fig. 7:**
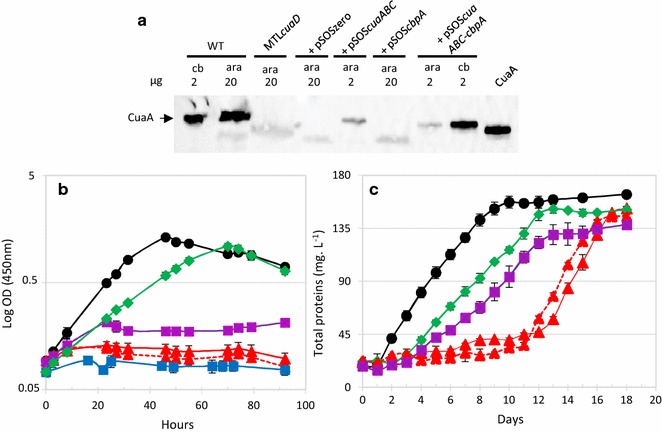
Growth and analysis of *R. cellulolyticum* wild-type and MTL*cuaD* complemented strains. **a** Western blot using anti-CuaA antiserum. The amounts of membrane fractions loaded in the gel are given in µg. Membrane fractions were isolated from an arabinose (ara) or a cellobiose (cb) grown culture. **b**, **c** Growth curve of different strains grown in minimal medium containing 2 g L^−1^ cellobiose (**b**) or 5 g L^−1^ cellulose (**c**). The strains are WT strain (black), MTL*cuaD* strain (red); MTL*cuaD* strain carrying an empty vector (red-dotted line), MTL*cuaD* strain carrying pSOS-*cbpA* (blue), MTL*cuaD* strain carrying pSOS*ABC (*purple), MTL*cuaD* strain carrying pSOS*ABC*-*cbpA* (green). Experiments were performed in triplicate and bars indicate the standard deviation

Western blot analyses confirmed the presence of CuaA in the mutant strains transformed with either pSOS*cuaABC* or pSOS*cuaABC*-*cbpA* (Fig. 7a). On arabinose, the growth of the transformed *R. cellulolyticum* mutant strains was not affected by the presence of the vectors compared to the wild-type strain (see Additional file 5). As expected on cellobiose, the mutant strain containing the control vector failed to grow, but complementation with the vector pSOS*cuaABC*-*cbpA* restored the growth although with a longer generation time (18 h) compared to the wild-type strain. The growth of the mutant strain complemented with the vector carrying only the genes encoding the ABC transporter (pSOS*cuaABC*) could not exceed a final OD value of 0.2 thus showing that the presence of the ABC transporter without CbpA cannot restore the wild-type phenotype. In this strain, cellobiose might be imported but is not optimally catabolized in the absence of CbpA thus leading to a very limited growth on the disaccharide. These results establish that CbpA plays a critical role during the growth on cellobiose. Complementation with the vector pSOS*cbpA* failed to restore any growth on cellobiose, thereby indicating that the cells cannot import cellobiose in the absence of the Cua ABC transporter. Altogether, these results indicate that in *R. cellulolyticum,* the ABC transporter and CbpA, whose gene expression is induced by the three-component system, are essential for both uptake and utilization of cellobiose. Nevertheless, it cannot be ruled out that the signalization mediated by *cuaDSR* may regulate the expression of other genes involved in cellobiose metabolism.

The degradation of cellulose performed by the cellulosomes releases cellobiose and longer cellodextrins. On this substrate, the mutant strain carrying the control vector behaved exactly like the untransformed mutant strain: after a slow growth of 12 days during which cellobiose accumulation was shown in the culture supernatant, the MTL*cuaD* (pSOSzeroTm) adapts and starts to consume rapidly cellobiose, to grow fast, and to produce CuaA (Figs. 4b, 7). This result highlights the importance of Cua ABC transporter during the growth on cellulose. As it was observed on cellobiose, the growth of the mutant strain carrying the vector pSOS*cuaABC*-*cbpA* is restored on cellulose although the growth is slightly delayed compared to the wild-type strain. As for the wild-type strain, cellobiose did not accumulate in the supernatant during the culture on cellulose of the MTL*cuaD*(pSOS*cuaABC*-*cbpA*) strain (see Additional file 6). The functional complementation of the mutant strain with only the ABC transporter also restored the growth on cellulose, although the final protein content at the stationary phase reached a lower value compared to wild-type and mutant MTL*cuaD* (pSOS*cuaABC*-*cbpA*) strains (Fig. 7). This result markedly differed from the data obtained for the same strain on cellobiose (nearly no growth). It could be explained by the fact that the ABC transporter can also import longer cellodextrins since CuaA was shown to bind to G2–G5. In this case, the imported longer cellodextrins may support the observed growth of the MTL*cuaD* (pSOS*cuaABC*) strain on cellulose, though the action of other intracellular enzyme(s) than CbpA, like dextrins hydrolases or phosphorylases.

On cellulose, the MTL*cuaD* (pSOS*cbpA*) strain started to grow 5 days later than the mutant strain, suggesting that an adaptation also occurred in this strain. The extended slow growth phase observed is probably attributable to the overproduction of CbpA in MTL*cuaD* (pSOS*cbpA*) strain that may reduce the fitness of the strain during this initial growth phase on cellulose.

## Discussion

Similar combined signalization and transport systems are described in other bacteria [25–28]. In Gram-negative bacteria, such genes clusters encode only one SBP that is implicated in both signalization and transport purposes, whereas in Gram-positive bacteria two SBPs are encoded like in *R. cellulolyticum*. In *C.* *beijerinckii*, each SBP was shown to be specifically dedicated for either signalization or transport [27]. In *R. cellulolyticum*, the studied SBPs also appear to have different functions: CuaA operates with the ABC transporter channel and helps to collect a large set of cellodextrins with a moderate affinity. In contrast, CuaD is probably committed to signalization since it was shown to bind specifically to cellobiose with a very high affinity and is genetically associated with the two-component system, thus forming the third subunit of a three-component system. CuaD would therefore sense the presence of cellobiose which is the shortest and most abundant degradation product released from cellulose by the cellulosomes in vitro [4].

**Fig. 8:**
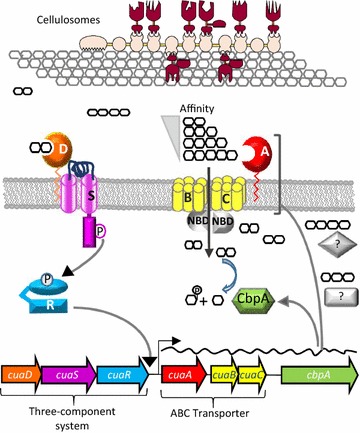
Model for cellodextrins utilization by *R. cellulolyticum.* The cellulosome is depicted in light (CipC)/dark (enzymes) brown, bound to cellulose chains, and releasing cellodextrins like cellobiose. The latter is recognized by CuaD (orange) bound to CuaS (purple) and signal is transmitted to the regulator CuaR (light blue) which subsequently induces the expression of the genes *cuaABC* and *cbpA*. The ABC transporter captures a large range of cellodextrins from the environment and transport them in the cytoplasm where the cellobiose phosphorylase CbpA cleaves the cellobiose in glucose and G1-P. Other cellodextrins might be degraded by other unknown cellodextrin phosphorylases or β-glucosidase. Unknown proteins like NBD and enzymes degrading intracellular dextrins are depicted in gray

Altogether, the data reported in the present study enable to propose the model depicted in Fig. 8. During the growth on cellulose, the cellulosomes secreted by *R. cellulolyticum* release a mix of cellodextrins from the substrate, among which cellobiose is detected by the solute binding protein CuaD of the three-component system CuaDSR. This signalization system is believed to transduce this signal via the sensor CuaS to the regulator CuaR which in turn would activate the transcription of the genes *cuaABC* and *cbpA* encoding the ABC transporter and the cellobiose phosphorylase CbpA, respectively. The ABC-transporter would then import the various released cellodextrins. The *cua* gene cluster does not encode any ABC transporter ATPase but the corresponding gene could be located elsewhere on the chromosome as formerly observed for other Gram-positive bacteria [29–32]. For instance, in *Bacillus subtilis*, the gene encoding the ABC ATPase MsmX was reported to be encoded in the genome by an isolated gene, and the corresponding protein was found to energize several ABC transporters dedicated to oligosaccharides uptake [30]. In this respect, an orthologous gene to *msmX* was identified in the genome of *R. cellulolyticum* at locus Ccel_2909. The gene product shares 65% homology with MsmX, and could energize the cellobiose/cellodextrin ABC transporter. This NBD could also energize other ABC transporters like the xyloglucan dextrins ABC transporter recently described, for which no ABC ATPase-encoding gene was found in the cluster encoding the system [3]. After uptake, CbpA would convert specifically cellobiose into glucose and glucose-1-P, as it was reported for other reported cellobiose phosphorylases [33, 34], whereas the degradation of larger imported cellodextrins (G3–G5) would require other enzyme(s). Four genes encoding putative cytoplasmic GH94 cellodextrin phosphorylases (at loci Ccel_1439, Ccel_2354, Ccel_3412), or β-glucosidases GH1 (Ccel_1139 and Ccel_0374) could be involved in this process [4, 35].

In cellulolytic bacteria such as *C. thermocellum* or *Ruminococcus albus*, phosphorolytic activity was however reported to dominate hydrolytic activity [16, 18]. As the phosphorolytic cleavage mechanism saves ATP during sugar catabolism, such process ought to be especially important for obligate anaerobic cellulolytic bacteria which grow with low ATP yields and need in addition to synthesize and secrete numerous cellulases to achieve an efficient degradation of the cellulose. From an energetic point of view, ABC transporter systems are assumed to require more energy to import carbohydrate compared to PTS uptake systems, which are not found in *R. cellulolyticum* [10]. Nevertheless, the higher cost in ATP to transport cellodextrins through ABC transporters may be balanced by the import of long cellodextrins which advantageously deliver more glucose units than short cellodextrins for the same cost, as it occurs in *C. thermocellum* [36]. Another advantage of the ABC transporters is that they display high uptake efficiency at low substrate concentration in the medium, thanks to the presence of the solute binding protein [20]. In *R. cellulolyticum*, the capture and import of cellodextrins is indeed rather efficient since nearly no soluble sugars are detected in the culture supernatant of the wild-type strain during the growth on cellulose. This efficient transport system might therefore contribute to increase the efficiency of the cellulolysis by preventing feedback inhibition of the cellulases by cellodextrins. In addition, the efficiency of the ABC transporter may help to reach the concentration threshold of intracellular cellobiose needed to thermodynamically favor phosphorolysis by CbpA [37].

**Fig. 9:**
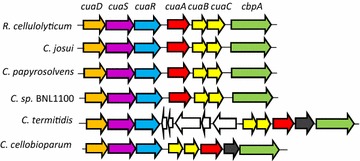
Similar *cua* gene clusters found in other sequenced cellulolytic bacteria. Each color corresponds to a gene function in different genomes as found in *cua* gene cluster in *R. cellulolyticum*. Additional genes encoding LacI-like regulator (black) or unrelated genes (white) are found in some genomes

Overall, the CuaABC transporter seems well adapted to anaerobic degradation of cellulose in *R. cellulolyticum* by scavenging efficiently cellodextrins present at low concentrations during the degradation of cellulose, reducing feed-back inhibition of the cellulases, and ensuring a requisite flow of substrate into the cell able to support phosphorolysis reaction. It is therefore not surprising that similar gene clusters are found in other anaerobic and cellulolytic bacteria whose genome was sequenced (Fig. 9). Recently, to produce cellulosic biofuel, synthetic biology strategies aimed to make yeasts and *E. coli* strains able to use cellobiose rather than glucose [38]. This was done through the acquisition of intracellular β-glucosidase, cellobiose phosphorylase, and/or cellobiose transporters from heterologous organisms [37–40], but none of the selected transporters used were ABC transporters. The efficient and energy-saving system of the conjugated transport/phosphorolysis described in the present study was shown to be well adapted to the low concentrations of soluble sugars released during cellulolysis, and to provide high enough intracellular sugar concentration for efficient phosphorolysis. This system adapted to cellulolytic life-style could therefore represent an attractive way to engineer strains for biofuels or chemicals production from ligno-cellulosic substrates.

## Conclusions

The present study identified a three-component signalization system inducing the expression of genes encoding an ABC transporter and a cellobiose phosphorylase. Through the study of a mutant strain and its derivatives, the genes encoding the ABC transporter and the cellobiose phosphorylase were shown to be essential for the growth of *R. cellulolyticum* on cellobiose and cellulose. This uptake and intracellular degradation of cellobiose system appears to be the major cellobiose utilization pathway in *R. cellulolyticum*. It seems well adapted to cellulolytic life-style and could be used to engineer new strain to produce biofuels and chemicals of interest from cellodextrins.

## Methods

### Strains, media and vectors

Strains used in this study are reported in Additional file 7. *Escherichia coli* strains were grown at 37 °C in Luria–Bertani medium supplemented with appropriate antibiotic (100 µg mL^−1^ of ampicillin or 35 µg mL^−1^ chloramphenicol). *R. cellulolyticum* H10 ATCC 35319 [41] and mutants were grown anaerobically at 32 °C on basal medium [1] or minimal medium (basal medium without yeast extract), supplemented with either 2 g L^−1^ cellobiose, arabinose, or 5 g L^−1^ Sigmacell20 (Sigma-Aldrich, Saint Louis, MO). When necessary, thiamphenicol (5 µg mL^−1^) or erythromycin (2.5 µg mL^−1^) was added to the medium. Colonies of recombinant *R. cellulolyticum* mutant strains were isolated under the anaerobic atmosphere of a glove box as described previously [24]. Growth on soluble substrate was followed by monitoring OD at 450 nm over time (arabinose, cellobiose). When cultured on 5 g L^−1^ Sigmacell, growth measurements were based on protein content quantification as described previously [42].

Vectors and primers used in this study are reported in Additional files 7 and 8, respectively.

Sequencing of strains was performed from genomic DNA by GATC (Konstanz, Germany).

### Analysis of cellulose- and soluble cellodextrin-content during growth

During growth, samples of culture were harvested and centrifuged. The cellulose pellets and the soluble sugars present in the supernatants were analyzed using high-pressure anion exchange chromatography coupled with pulsed amperometric detection (HPAEC-PAD). After centrifugation, the pellets of the different growth samples were resuspended in 500 μL of 12 M H_2_SO_4_ and incubated for 1 h at 37 °C under shaking. For each sample, 20 µL was diluted into 220 μL of distilled water and autoclaved for 1 h at 120 °C. After cooling down, 50 μL of 10 M NaOH was added and the sample was centrifuged at 10,000*g* for 10 min at room temperature. 10 µL of supernatant was mixed with 190 μL of distilled water and 50 μL of 0.5 M NaOH. Glucose was quantified by high-pressure anion exchange chromatography using a Dionex ICS 3000 (Sunnyvale, CA) equipped with a pulsed amperometric detector (HPAEC-PAD). 25 µL was applied to a Dionex CarboPac PA1 column (4 × 250 mm) preceded by the corresponding guard column (4 × 50 mm). Sugars were eluted with the buffers 0.1 M NaOH and 0.5 M sodium acetate + 0.1 M NaOH as the eluants A and B, respectively, using for glucose the multi-step procedure: isocratic separation (5 min, 95% A + 5% B), column wash (2 min, 99% B), and subsequent column equilibration (2.5 min, 95% A + 5% B). Injection of samples containing glucose (Sigma) at known concentrations (5–100 nM) was used to quantify the glucose content.

The culture supernatants were analyzed similarly except that 20 µL of supernatant was mixed with 190 μL of distilled water and 50 μL of 0.5 M NaOH, prior HPAEC-PAD analyses. In that case, sugars were eluted using the multi-step procedure: isocratic separation (5 min, 95% A + 5% B), separation gradient (8 min, 10 to 37% B), column wash (2 min, 99% B), and subsequent column equilibration (2.5 min, 95% A + 5% B). Injections of samples containing glucose, cellobiose (Sigma), cellotriose, cellotetraose, and cellopentaose (Megazyme) at known concentrations (5–100 nM) were used to identify and quantify the cellodextrins present in the culture supernatants.

### Construction of *cuaD* mutations in *R. cellulolyticum*

Gene inactivation in *R. cellulolyticum* was performed using the ClosTron technology as described by Heap et al. [23] with minor modifications to create the pMTL007*cuaD* and pMTL007*cuaA* [23, 24]. The integration sites in the target genes and the primers used to retarget the Ll.LtrB intron in the pMTL007 [IBS, EBS1d and EBS2] (see Additional file 8) were generated by the Perutka algorithm (http://ClosTron.com). Specific *cuaD* and *cuaA* target primers were used to produce a fragment by overlapping PCR using pMTL007 as the matrix. The fragments and the recipient pMTL007 were subsequently digested by *Bsr*GI and *Hin*dIII and ligated, creating the pMTL007*cuaD* and pMTL007*cuaA* to be used for electro-transformation of *R. cellulolyticum*. Electro-transformation was performed as previously described [43, 44]. The modified strain interrupted in the gene at locus Ccel_2115 was called MTL*cuaD*.

### Complementation of MTL*cuaD* mutant

As the MTL*cuaD*-mutated strain already contains erythromycin resistance brought by the insertion in the genome, we used a thiamphenicol-resistant vector pSOSzeroTm [45]. This vector was digested using *Sal*I and ligated with the expression cassette obtained from pSOS954 digested by the same enzyme. The resulting *E. coli*–*R. cellulolyticum* shuttle expression vector called pSOS956 was then used to clone the genes *cuaABC*, *cuaABC*-*cbpA*, and *cbpA*. Amplicons were obtained by PCR using genomic DNA of *R. cellulolyticum* as the template and the primers pairs 2112BamDir/2110NarRev, and 2109BamDir/2109NarRev. Corresponding amplicons were digested with *Bam*HI and *Nar*I, and ligated to pSOS956 similarly digested to give the vectors pSOS*cuaABC* and pSOS*cbpA*. To construct the vector pSOS*cuaABC*-*cbpA*, the PCR product obtained using the primers pair 2109NarDir/2109NarRev on genomic DNA from *R. cellulolyticum* was digested with *Nar*I and ligated in the *Nar*I-digested pSOS*cuaABC*. This construction brings *cbpA* downstream of *cuaABC* under the control of the same promotor, but it removes the intergenic region found naturally upstream of *cbpA*. The resulting vectors were called pSOSzeroTm, pSOS*ABC*, pSOS*ABC*-*cbpA*, and pSOS*cbpA*, and were transferred in the MTL*cuaD* mutant strain as previously described [43, 44].

### RNA preparation and reverse transcription

*Ruminiclostridum cellulolyticum* was grown in minimal medium supplemented with arabinose (2 g L^−1^), cellobiose (2 g L^−1^), or cellulose (5 g L^−1^). Samples were harvested at mid or late-exponential phase of growth (8000 g 10 min) and frost at − 80 °C. Total RNAs were isolated from the pellet using the Maxwell® 16 LEV simplyRNA Blood Kit (Promega) according to the manufacturer’s instructions and an extra TURBO DNase digestion step to eliminate the contaminating DNA. The RNA quality was assessed by an Experion chip (Bio-Rad), and the absence of DNA contamination was confirmed by PCR. RNA was quantified spectrophotometrically at 260 nm (NanoDrop 1000; Thermo Fisher Scientific). For cDNA synthesis, 1 µg total RNA and 0.5 μg random primers (Promega) were used with the GoScript™ Reverse transcriptase (Promega) according to the manufacturer instruction.

### Quantitative real-time-PCR for transcriptional analyses

Intergenic links and qPCR analysis were performed on cDNA using primers listed in Additional file 8. Intergenic links were analyzed as previously described [45]. qPCR was carried out on a CFX96 real-time PCR detection system (Biorad, France) and the software Manager CFX 3.1 using RNA16S gene as a reference for normalization. For qPCR, the reaction volume was 15 μL and the final concentration of each primer was 0.5 μM. The cycling parameters of the qPCR were 98 °C for 2 min, followed by 45 cycles of 98 °C for 5 s, 56 °C for 10 s, 72 °C for 1 s. A final melting curve from 65 to 95 °C is added to determine the specificity of the amplification. To determine the amplification kinetics of each product, the fluorescence derived from the incorporation of EvaGreen into the double-stranded PCR products was measured at the end of each cycle using the SsoFast EvaGreen Supermix 2X Kit (Bio-Rad, France). The results were analyzed using Bio-Rad CFX Manager software, version 3.0 (Bio-Rad, France). For each point, a technical duplicate was performed. The amplification efficiencies for each primer pairs comprised between 80 and 100%.

### Fluorescence measurements

The gene *cuaR* was amplified by PCR using the forward and the reverse primers pBadRegFLEcoD and pBadRegPstR, and the amplicon was digested by *Eco*RI and *Pst*I before ligation with EcoRI-PstI linearized pBAD24 [46] to give pBAD-*cuaR*. Intergenic regions (IG) located upstream of the genes at the locus Ccel_2116, *cuaD*, *cuaA*, and *cbpA* were amplified using forward and reverse primers pairs IG1gfpXhoD/IG1gfpBamR, IG2gfpXhoD/IG2gfpBamR, IG3gfpXhoD/IG3gfpBamR, IG4gfpXhoD/IG4gfpBamR respectively. Amplicons were digested using *Xho*I and *Bam*HI, and ligated to linearized pUA66 vector allowing transcriptional fusions with *gfpmut2* gene (see Additional file 8) [47]. The vectors pBAD24, pBAD24*cuaR*, and pUA66, pUA66-IG1, pUA66-IG2, pUA66-IG3, and pUA66-IG4 were transferred in MG1665 *E. coli* strain for fluorescence measurements. Recombinant MG1655 *E. coli* cells containing a pBAD24 and a pUA66 derivative vector were grown overnight in minimal medium M9, 0.2% casamino acids (Gifco, USA), with 100 µg mL^−1^ ampicillin and 25 µg mL^−1^ kanamycin. Cultures were diluted in the same medium at an OD of 0.1 in a 96-well plate (Greiner, Germany). Either 0.1% arabinose or the same volume of water was added, and the plate was incubated at 37 °C overnight under gentle shaking. OD at 600 nm and GFP fluorescence (488 nm and 521 nm excitation and emission wavelengths, respectively) were read using a Tecan i-control™ 1.9.17.

### Cloning of the genes encoding rCuaA and rCuaD and rCbpA in *E. coli*

Recombinant mature proteins rCuaA, rCuaD, and rCbpA were designed to contain 6 histidines residues at their N-terminus (rCuaA, rCuaD) or at the C-terminus (rCbpA). All genes were amplified by PCR using the genomic DNA of *R*. *cellulolyticum* and the forward and reverse primers pairs CuaANdeID/CuaAXhoIR, CuaDNdeID/CuaDXhoIR, CbpANdeID/CbpAXhoIR for the production of rCuaA, rCuaD, and rCbpA, respectively. The amplicons were digested with *Nde*I and *Xho*I and cloned in a *Nde*I–*Xho*I linearized pET22b(+), thereby generating the pET-*cuaA* pET-*cuaD*, or digested with *Nco*I and *Xho*I and cloned into a *Nco*I-*Xho*I linearized pET28a(+) generating pET-*cbpA*. The generated plasmids were used to transform BL21(DE3) strain to produce the corresponding recombinant proteins.

### Production and purification of the recombinant proteins

Recombinant *E. coli* BL21(DE3) containing the vectors pET*cuaA*, pET*cuaD*, or pET*cbpA* were grown at 37 °C under shaking to an optical density at 600 nm of 1.0, IPTG was added to a final concentration of 200 µM, and the cultures were incubated overnight under shaking at 16 °C. The cells were then harvested by centrifugation for 15 min at 5000*g* and broken in a French press before to proceed to a purification through affinity chromatography and anion exchange chromatography as described before [24]. The purified protein concentration was determined by measuring the absorbance at 280 nm.

### PAGE and Western blot analysis

Sodium dodecyl sulfate–polyacrylamide gel electrophoresis (SDS-PAGE) was performed as previously described [24]. Primary antibodies were obtained using the purified recombinant rCuaA and rCuaD proteins (Eurogentec, France).

### Preparation of membrane proteins from *R. cellulolyticum*

*Ruminiclostridum cellulolyticum* cells were harvested from a minimal medium grown culture (90 mL) at mid exponential stage of growth (7000*g*, 15 min, 4 °C). Pelleted cells were broken in buffer containing 50 mM KH_2_PO_4_, 150 mM NaCl, pH7, using a French press. After centrifugation (45,000*g*, 30 min), the pellets containing the membranes were collected in the same buffer containing 1% SDS. Protein content was determined using the Lowry method [48]. Membrane proteins (5 µg) were loaded on a SDS-PAGE to check the quality of the samples (Additional file 9).

### Isothermal titration calorimetry (ITC)

Ligand binding parameters were measured using a Microcal iTC200 calorimeter (Malvern) at 20 °C in 150 mM, NaCl, 25 mM HEPES, pH 7.0. Concentrated solutions of glucose, arabinose, cellobiose (Sigma, St Louis MO) and cellotriose, cellotetraose, cellopentaose (Megazyme, Ireland) were diluted to a concentration varying between 100 and 600 µM. The protein concentration in the cell (200 µL) was diluted in the same buffer to a concentration varying between 10 and 60 µM. Data were fitted by nonlinear regression using a single-site model in the Origin 7 software package.

### Phosphorylase activity measurement

rCbpA (0.5 µM) was incubated with 0.05–9 mM cellobiose (Sigma) in 25 mM phosphate buffer pH 6 containing 0.01% (w/v) NaN_3_ in a final volume of 200 µL at 37 °C during 2 min. Then 50 µL of 0.5 M sodium hydroxide was added prior to analysis by HPAEC-PAD, using the same procedure as for cellodextrin content analyses, except that various concentrations of glucose, cellobiose, and α-d-glucose-l-phosphate (Sigma) were used to identify and quantify the released sugars. Activity of rCbpA on 1 mM cellotriose, cellotetraose, and cellopentaose was assayed with 1 µM of enzyme and incubation times up to 120 min at 37 °C were used.

### Bioinformatics analysis

Nucleotide sequences analysis were performed using BPROM, FindTerm (http://www.softberry.com) or ARNold (http://rna.igmors.u-psud.fr). Amino acid sequence analysis were performed using on line tools: Signal P, LipoP (http://www.cbs.dtu.dk), BLAST (https://blast.ncbi.nlm.nih.gov/Blast.cgi) and ClustalW2 (http://www.ebi.ac.uk).

## Additional files

**Additional file 1.** Representative ITC data of the interactions of CuaA and CuaD with cellodextrins. Integrated binding heats of interactions of CuaA and CuaD with cellobiose are shown along with the binding parameters of the interaction.

**Additional file 2.** qPCR analysis of mRNA produced by WT strain. qPCR was performed on cDNA prepared on total RNA that was extracted from cultures of *R. cellulolyticum* grown on minimal medium containing 2 g.L^−1^ arabinose, cellobiose or 5 g.L^−1^ cellulose.

**Additional file 3.** Comparison of *cua* genes expression level between WT and MTL*cuaD* mutant strain grown on arabinose. qPCR was performed on cDNA prepared on total RNA extracted from cultures of *R. cellulolyticum* WT and MTL*cuaD* strains grown on minimal medium containing 2 g.L^−1^ arabinose. Expression of each gene of the mutant strain MTL*cuaD* is presented compared to the WT gene expression normalized to 1.

**Additional file 4.** Mutations found in the sequence of the adapted MTL*cuaD* mutant strain. The table reports all single-nucleotide polymorphism (SNP) and insertions/deletions mutations (In/Del) type which were common to two individual MTL*cuaD* mutant strains clones and absent from the MTL*cuaD* mutant strain.(white background), and the mutations that were found only in the mutant strain (grey background).

**Additional file 5.** Growth of the MTL*cuaD* complemented strain on arabinose. The data shows the growth of WT, MTL*cuaD,* MTL*cuaD*(pSOSzero-Tm), MTL*cuaD*(pSOS*cbpA*), MTL*cuaD*(pSOS*cuaABC*), MTL*cuaD*(pSOS*cuaABC*-*cbpA*) strains on minimal medium containing 2 g.L^−1^ arabinose.

**Additional file 6.** Growth of two complemented MTL*cuaD* strains on cellulose. The data shows the growth, the degradation of cellulose and the concentration of cellobiose in the supernatant of the culture of the strains MTL*cuaD* (pSOS*cuaABC*) and MTL*cuaD* (pSOS*cuaABC*-*cbpA*) on minimal medium containing 5 g.L^−1^ cellulose.

**Additional file 7.** Bacterial strains and vectors used.

**Additional file 8.** Primer used.

**Additional file 9.** Analysis of membrane protein samples. 5 µg of the membrane fractions prepared from different strains and growth conditions are loaded on SDS-PAGE stained with Coomassie Blue before to be analyzed by Western blot.

## Abbreviations

SBP: solute binding protein
TMD: transmembrane domain
NBD: nucleotide binding domain
