# Supplementary material for: A seven-gene cluster in Ruminiclostridium cellulolyticum is essential for signalization, uptake and catabolism of the degradation products of cellulose hydrolysis
Source: Biotechnol Biofuels. 2017 Oct 30;10:250. doi: 10.1186/s13068-017-0933-7 (PMC5663094; doi:10.1186/s13068-017-0933-7)
Supplement: Supplementary file 1 — Additional file 1. Representative ITC data of the interactions of CuaA and CuaD with cellodextrins. Integrated binding heats of interactions of CuaA and CuaD with cellobiose are shown along with the binding parameters of the interaction. [file 13068_2017_933_MOESM1_ESM.pdf]

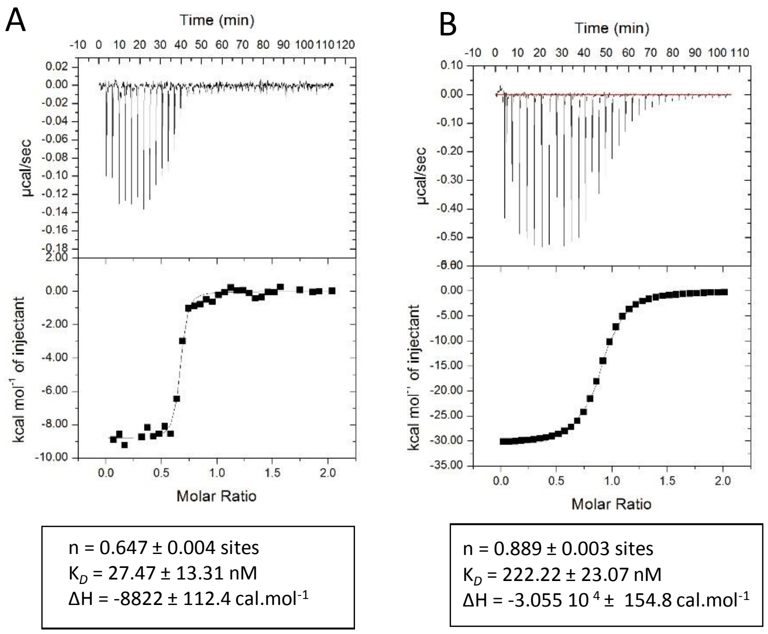

Additional file 1. Representative ITC data of the interactions of CuaA and CuaD with cellodextrins

Interactions of CuaA (A) and CuaD (B) with cellobiose are shown. The top half of each panel represents the raw binding heats and the lower half represents the integrated binding heats minus the dilution control heats fitted to a single-site binding model. Insert presents the binding parameters, with n designating the number of binding sites on the protein.
