# Supplementary material for: A seven-gene cluster in Ruminiclostridium cellulolyticum is essential for signalization, uptake and catabolism of the degradation products of cellulose hydrolysis
Source: Biotechnol Biofuels. 2017 Oct 30;10:250. doi: 10.1186/s13068-017-0933-7 (PMC5663094; doi:10.1186/s13068-017-0933-7)
Supplement: Supplementary file 2 — Additional file 2. qPCR analysis of mRNA produced by WT strain. qPCR was performed on cDNA prepared on total RNA that was extracted from cultures of R. cellulolyticum grown on minimal medium containing 2 g.L−1 arabinose, cellobiose or 5 g.L−1 cellulose. [file 13068_2017_933_MOESM2_ESM.pdf]

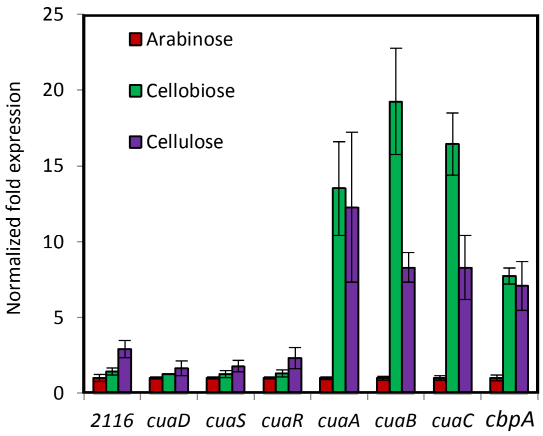

Additional file 2. qPCR analysis of mRNA produced by WT strain
Total RNA was extracted from cultures of *R. cellulolyticum* grown in minimal medium supplemented with 0.2% arabinose, cellobiose or 0.5% cellulose as the sole carbon source. Normalization was performed using the RNA16 gene. Error bars indicate the standard deviation of three independents experiments.
