# Supplementary material for: A seven-gene cluster in Ruminiclostridium cellulolyticum is essential for signalization, uptake and catabolism of the degradation products of cellulose hydrolysis
Source: Biotechnol Biofuels. 2017 Oct 30;10:250. doi: 10.1186/s13068-017-0933-7 (PMC5663094; doi:10.1186/s13068-017-0933-7)
Supplement: Supplementary file 3 — Additional file 3. Comparison of cua genes expression level between WT and MTLcuaD mutant strain grown on arabinose. qPCR was performed on cDNA prepared on total RNA extracted from cultures of R. cellulolyticum WT and MTLcuaD strains grown on minimal medium containing 2 g.L−1 arabinose. Expression of each gene of the mutant strain MTLcuaD is presented compared to the WT gene expression normalized to 1. [file 13068_2017_933_MOESM3_ESM.pdf]

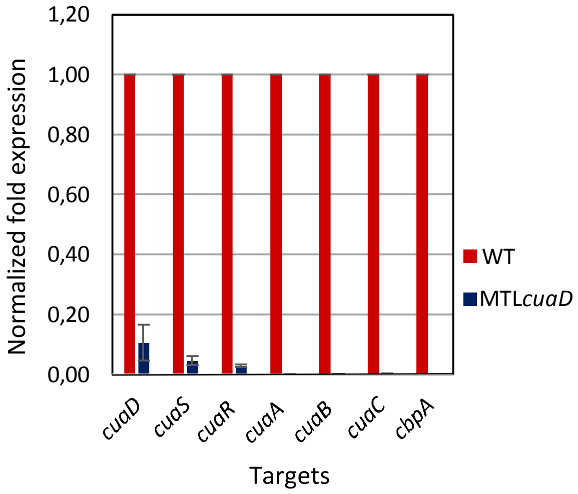

Additional file 3. Comparison of *cua* genes expression level between WT and MTL*cuaD* mutant strain grown on arabinose. qPCR was performed on cDNA prepared on total RNA extracted from cultures of *R. cellulolyticum* WT and MTL*cuaD* strains grown on minimal medium containing 2 g.L^{-1} arabinose. Expression of the gene of the mutant strain MTL*cuaD* is presented compared to the WT gene expression normalized to 1.
