# Supplementary material for: A seven-gene cluster in Ruminiclostridium cellulolyticum is essential for signalization, uptake and catabolism of the degradation products of cellulose hydrolysis
Source: Biotechnol Biofuels. 2017 Oct 30;10:250. doi: 10.1186/s13068-017-0933-7 (PMC5663094; doi:10.1186/s13068-017-0933-7)
Supplement: Supplementary file 4 — Additional file 4. Mutations found in the sequence of the adapted MTLcuaD mutant strain. The table reports all single-nucleotide polymorphism (SNP) and insertions/deletions mutations (In/Del) type which were common to two individual MTLcuaD mutant strains clones and absent from the MTLcuaD mutant strain.(white background), and the mutations that were found only in the mutant strain (grey background). [file 13068_2017_933_MOESM4_ESM.pdf]

Single-nucleotide polymorphism in the sequence of both adapted strains compared to the original mutant MTL*cuaD* strain							
Position	Reference base	Observed base	Amino acid change	Effect	Functional class	Localization	Predicted gene product
455589	A	T	NA	Upstream	None	Bw Ccel_R0007 / Ccel_R0008	rRNA-16S / tRNA-Ala
2127097	G	T	NA	Upstream	None	Bw Ccel_1774 / Ccel_R0045	ATP dependant helicase-nuclease subunit A / tRNA-Gln
2127098	C	T	*1249	Synonymous_stop	Silent	Ccel_1774	ATP dependant helicase-nuclease subunit A
2606472	C	G	D380H	Non_synonymous_ Coding	Missense	Ccel_2228	Hypothetical protein
2914046	A	C	NA	Upstream	None	Bw Ccel_R0059 / Ccel_R0058	rRNA-16S / rRNA-23S
4055304	A	G	NA	Upstream	None	Bw Ccel_R0088 / Ccel_R0087	rRNA-16S / rRNA-23S
2316753	C	T	G263S	NON_SYNONYMOUS_ CODING	MISSENSE	Ccel_1956	cell wall associated hydrolase

Insertions/deletions in the sequence of both adapted strains compared to the original mutant MTL*cuaD* strain						
Position	Reference base	Observed base	Effect	Functional class	Localization	Gene product
455591	A	Act	Upstream	None	Bw Ccel_R0007 / Ccel_R0008	rRNA-16S / tRNA-Ala
455593	Tgg	T	Upstream	None	Bw Ccel_R0007 / Ccel_R0008	rRNA-16S / tRNA-Ala
2471924	Gga	G	Upstream	None	Bw Ccel_2112 / Ccel_2111	CuaA / CuaB

Additional file 4. Mutations found in the sequence of the adapted MTL*cuaD* mutant strain. The table reports all single-nucleotide polymorphism (SNP) and insertions/deletions mutations (In/Del) type which were common to two individual MTL*cuaD* mutant strains clones and absent from the MTL*cuaD* mutant strain.(white background), and the mutations that were found only in the mutant strain (grey background). (NA: not applicable ; *: stop codon; Bw: between)
