# Supplementary material for: A seven-gene cluster in Ruminiclostridium cellulolyticum is essential for signalization, uptake and catabolism of the degradation products of cellulose hydrolysis
Source: Biotechnol Biofuels. 2017 Oct 30;10:250. doi: 10.1186/s13068-017-0933-7 (PMC5663094; doi:10.1186/s13068-017-0933-7)
Supplement: Supplementary file 5 — Additional file 5. Growth of the MTLcuaD complemented strain on arabinose. The data shows the growth of WT, MTLcuaD, MTLcuaD(pSOSzero-Tm), MTLcuaD(pSOScbpA), MTLcuaD(pSOScuaABC), MTLcuaD(pSOScuaABC-cbpA) strains on minimal medium containing 2 g.L−1 arabinose. [file 13068_2017_933_MOESM5_ESM.pdf]

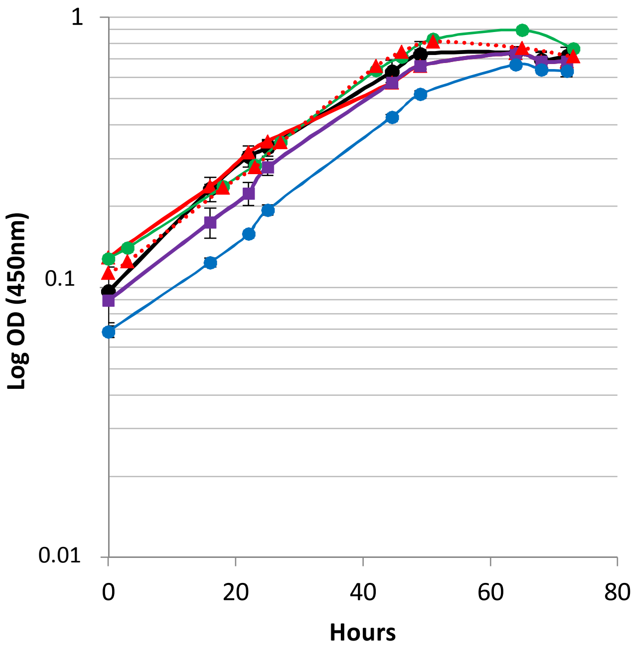

Additional file 5 : Growth of the MTL*cuaD* complemented strain on arabinose

The strains were grown on minimal medium containing 2 g.L^{-1} arabinose. WT strain (black), MTL*cuaD* strain (red); MTL*cuaD* strain carrying an empty vector (red dotted line); MTL*cuaD* strain carrying pSOS*cbpA* (blue); MTL*cuaD* strain carrying pSOS*ABC* (purple); MTL*cuaD* strain carrying pSOS*ABC*-*cbpA* (green). Experiments were performed in triplicate and bars indicate the standard deviation.
