# Supplementary material for: A seven-gene cluster in Ruminiclostridium cellulolyticum is essential for signalization, uptake and catabolism of the degradation products of cellulose hydrolysis
Source: Biotechnol Biofuels. 2017 Oct 30;10:250. doi: 10.1186/s13068-017-0933-7 (PMC5663094; doi:10.1186/s13068-017-0933-7)
Supplement: Supplementary file 6 — Additional file 6. Growth of two complemented MTLcuaD strains on cellulose. The data shows the growth, the degradation of cellulose and the concentration of cellobiose in the supernatant of the culture of the strains MTLcuaD (pSOScuaABC) and MTLcuaD (pSOScuaABC-cbpA) on minimal medium containing 5 g.L−1 cellulose. [file 13068_2017_933_MOESM6_ESM.pdf]

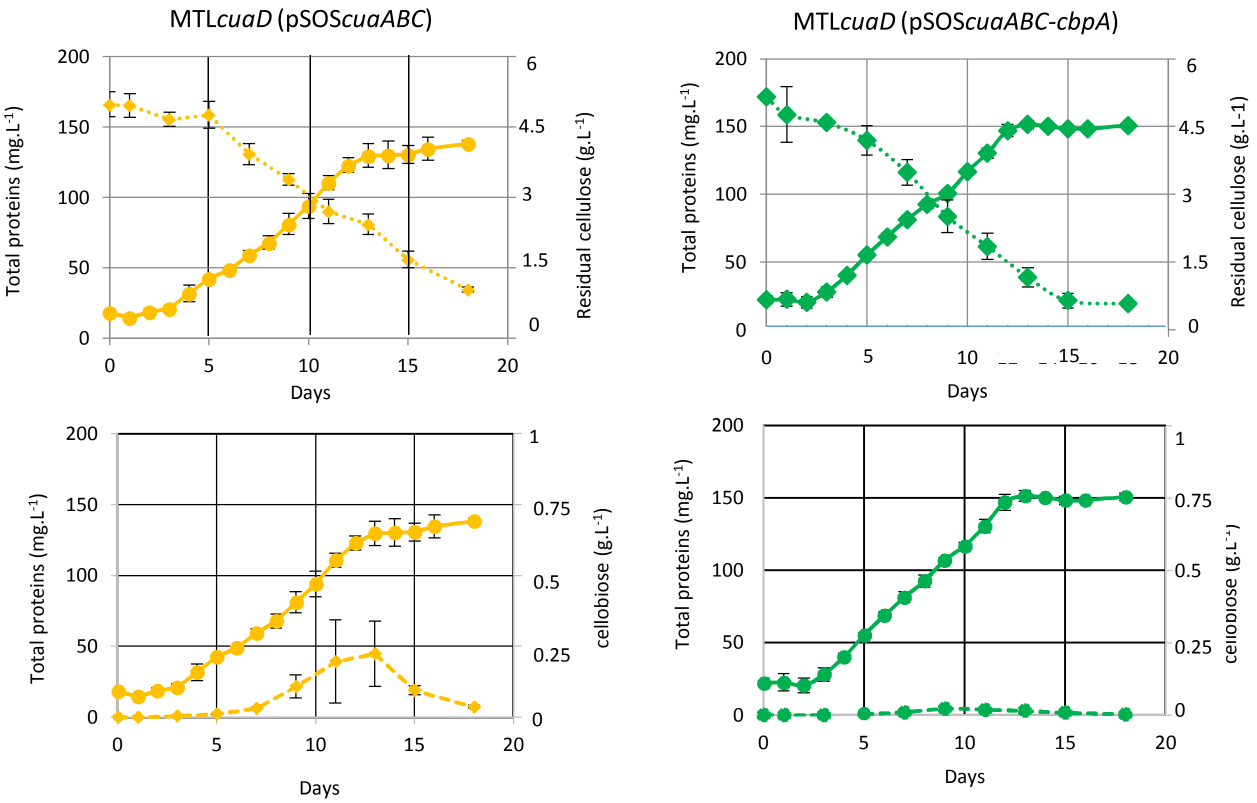

Additional file 6. Growth of two complemented MTL*cuaD* strains on cellulose

The strains MTL*cuaD* (pSOS*cuaABC*) and MTL*cuaD* (pSOS*cuaABC-cbpA*) were grown on minimal medium containing 5 $g.L^{-1}$ cellulose. Upper panels show the growth on cellulose (solid line) and the degradation of cellulose (dotted line). Lower panels show the growth on cellulose (solid line) and the concentration of cellobiose in the supernatant of the culture (dotted line).
