# Supplementary material for: A seven-gene cluster in Ruminiclostridium cellulolyticum is essential for signalization, uptake and catabolism of the degradation products of cellulose hydrolysis
Source: Biotechnol Biofuels. 2017 Oct 30;10:250. doi: 10.1186/s13068-017-0933-7 (PMC5663094; doi:10.1186/s13068-017-0933-7)
Supplement: Supplementary file 7 — Additional file 7. Bacterial strains and vectors used. [file 13068_2017_933_MOESM7_ESM.pdf]

Additional file_7. Bacterial strains and vectors used

Strain or plasmid	Relevant characteristics	Source or reference
E. coli DH5a	F^{-} *endA1 hsdR17*(rK^{-} mK^{+}) *supE44 thi-1* λ^{-} *gyrA96 relA1*Δ(*lacZYA argF*) *U169* (Φ80 *lacZ*ΔM15) *recA*	Roche Diagnostics
E. coli SG13009(pREP4)	F^{-} *his pyrD Δlon-100 rpsL* (pREP4)	Qiagen
E. coli BL21(DE3)	F^{-} *ompT hsdS* (rB^{-} mB^{-}) *gal dcm* (DE3)	Novagen
C. cellulolyticum H10	Wild-type, ATCC35519 DSM 5812	DSMZ
C. cellulolyticum MTL*cuaD*	ATCC35319, *cuaD* ::intron, Erm^{r}	This study
C. cellulolyticum MTL*cuaD* adapted	ATCC35319 derivative, *cuaD* ::intron, Erm^{r}, able to grow on cellobiose/cellulose	This study
pET22b(+) and pET28a(+)	*E. coli* expression vector, Amp^{r} and Kan^{r} respectively	Novagen
pET*cuaD*	pET22b+ derivative carrying the NdeI-XhoI fragment encoding mature CuaD	This study
pET*cuaA*	pET22b+ derivative carrying the NdeI-XhoI fragment encoding mature CuaA	This study
pET*cbpA*	pET28a+ derivative carrying the NdeI-XhoI fragment encoding CbpA	This study
pBAD24	*E. coli* expression vector, Kan^{r}	(46) gift M. Ansaldi
pBAD*cuaR*	pBAD24 derivative carrying the EcoRI-PstI fragment encoding CuaR	This study
pUA66	*E coli* (pSC101), Kan^{R}, *gfpmut2*	(47) gift M Ansaldi
pUA66-IG1	pUA66 derivative carrying the 580 bp XhoI-BamHI intergenic region upstream of the gene at the locus Ccel_2108	This study
pUA66-IG2	pUA66 derivative carrying the 423 bp XhoI-BamHI intergenic region upstream of *cuaD*	This study
pUA66-IG3	pUA66 derivative carrying the 535 bp XhoI-BamHI intergenic region upstream of *cuaA*	This study
pUA66-IG4	pUA66 derivative carrying the 924 bp XhoI-BamHI intergenic region upstream of *cbpA*	This study
pMTL007	*E. coli/Clostridium* shuttle vector (ColE1, pCB102)Ll.*ltr*Bintron (*erm*BtdRAM2) under the control of P*fac*, *ltr*A; Cm^{r}/Tm^{r}	(23)
pMTL007*cuaD*	pMTL007 derivative targeting *cuaD* (locus Ccel_2115)	This study
pSOSzero-Tm	*E. coli/Clostridium* shuttle vector (ColE1, pIM13); Ap^{r}, Cm^{r}/Tm^{r}	(45)
pSOS954	*E. coli/Clostridium* shuttle vector (ColE1, pIM13); Ap^{r},Em^{R}, containing an expression cassette controled by the *thiolase* gene promotor (P$_{thl}$) from *C. acetobutylicum*	(24)
pSOS956	pSOSzero-Tm derivative carrying the SalI-SalI expression cassette from *C. acetobutylicum* from pSOS954, Ap^{r}, Cm^{r}/Tm^{r}	This study
pSOS***cua****ABC*	pSOS956 carrying the BamHI-NarI fragment encoding full length *cuaA*, *cuaB*, *cuaC* genes, Ap^{r}, Cm^{r}/Tm^{r}	This study
pSOS***cua****ABC-cbpA*	pSOS956 carrying the BamHI-NarI fragment encoding full length *cuaA*, *cuaB*, *cuaC*, *cbpA* genes, Ap^{r}, Cm^{r}/Tm^{r}	This study
pSOS*cbpA*	pSOS956 carrying the BamHI-NarI fragment encoding full length *cbpA*, Ap^{r}, Cm^{r}/Tm^{r}	This study

Ap^{r}, ampicilline resistance ; Erm^{r}, erythromycin resistance ; Kan^{r}, kanamycine resistance ; Cm^{r}/Tm^{r}, chloramphenicol/thiamphenicol resistance
