# Supplementary material for: A seven-gene cluster in Ruminiclostridium cellulolyticum is essential for signalization, uptake and catabolism of the degradation products of cellulose hydrolysis
Source: Biotechnol Biofuels. 2017 Oct 30;10:250. doi: 10.1186/s13068-017-0933-7 (PMC5663094; doi:10.1186/s13068-017-0933-7)
Supplement: Supplementary file 8 — Additional file 8. Primer used. [file 13068_2017_933_MOESM8_ESM.pdf]

Additional file_8: Primer used

EXPERIMENT	NAME	SEQUENCE	FEATURES
**Targeted mutagenesis**	IBS*cuaD*_887	AAAAAAGCTTATAATTATCCTTAGGTATCATCTCAGTGCGCCCAGATAGGGTG	
	EBS1d*cuaD*_887	CAGATTGTACAAATGTGGTGATAACAGATAAGTCATCTCAACTAACTTACCTTTCTTTGT	
	EBS2*cuaD*_887	TGAACGCAAGTTTCTAATTTCGATTATACCTCGATAGAGGAAAGTGTCT	
	EBS universal	CGAAATTAGAAACTTGCGTTCAGTAAAC	
	IBS*cuaA*_89	AAAAAAGCTTATAATTATCCTTAGATGCCGAAGCAGTGCGCCCAGATAGGGTG	
	EBS1d*cuaA*_89	CAGATTGTACAAATGTGGTGATAACAGATAAGTCGAAGCAGTTAACTTACCTTTCTTTGT	
	EBS2*cuaA*_89	TGAACGCAAGTTTCTAATTTCGATTGCATCTCGATAGAGGAAAGTGTCT	
	IBS*cuaA*_786	AAAAAAGCTTATAATTATCCTTAGCCAGCCTTCCAGTGCGCCCAGATAGGGTG	
	EBS1d*cuaA*_786	CAGATTGTACAAATGTGGTGATAACAGATAAGTCCTTCCACATAACTTACCTTTCTTTGT	
	EBS2*cuaA*_786	TGAACGCAAGTTTCTAATTTCGATTCTGGCTCGATAGAGGAAAGTGTCT	
**Control of the mutation**	CuaD_734Dir	ATATGAAGGAACTTCACAAGATGC	
	CuaD_1187Rev	ACATGATCAGGGATTGCACAC	
**Production in *E. coli***	CuaDNdeID	AATTCAT**ATG**CACCACCACCACCACCACTGCAGCAGTGACAATTCG	NdeI is underlined, ATG boldface type
	CuaDXhoIR	AATTCTCGAGTTACTGCAATTTGTCTAATGCTTTTTCC	XhoI is underlined
	CuaANdeID	AATTCAT**ATG**CACCACCACCACCACCACTGTGGAAACACAGAGTCAGG	NdeI is underlined, ATG boldface type
	CuaAXhoIR,	AATTCTCGAGTTACTTCAATTCAGGAACATTTTTCTTC	XhoI is underlined
	2109Ncof	TTTTTTCC**ATG**GTGAAATACGGTTTCTTTGATGATA	NcoI is underlined, ATG boldface type
	2109Xhor	TTTTTTCTCGAGTCCCATTATTACTTCGACCTGATG	XhoI is underlined
**Fluorescence studies**	pBadRegFLEcoD	TTACGAATTCACCATGTACAAGGTTCTAATAATTGATG	EcoRI is underlined
	pBadRegPstR	ATATCTGCAGTTAATAACAACTATCTCTGTATTCCG	PstI is underlined
	IG1gfpXhoID	AAGCCTCGAGCACCTGTAAGTACAACTCATGTTATATC	XhoI is underlined
	IG1gfpBamR	AATAGGATCCTTACGCATAAGCCCTCCTG	BamHI is underlined
	IG2gfpXhoD	AAGGCTCGAGGGACCGCCTGTCAGAAG	XhoI is underlined
	IG2gfpBamR	AATAGGATCC CACTGCTGCAAGAAACTG	BamHI is underlined
	IG3gfpXhoD	AAGGCTCGAGAAGGCAAAAGAACTTCTAAAGG	XhoI is underlined
	IG3gfpBamR	AATAGGATCCATTCCCAGTACCATAACGCC	BamHI is underlined
	IG4gfpXhoD	AAGGCTCGAGAGCAAACTATGGTGCCAG	XhoI is underlined
	IG4gfpBamR	AATCGGATCCAGGATAAGGTGTTTTAGGTGTTG	BamHI is underlined

EXPERIMENT	NAME	SEQUENCE	FEATURES
**Complementation studies**	2112BamDir	ATTAAGGATCC**A**GAATTTAAAAGGAGGGATTAAAATGTTTAAAAAGGTAATAGCTTCTG	BamHI is underlined
	2110NarRev	CCTTGGCGCCTTAGCCTTTTACAGACCCTGC	NarI is underlined
	2109NarRev	AATTGGCGCCTTATCCCATTATTACTTCGACCTG	NarI is underlined
	2109NarDir	TTAAGGCGCCAGAATTTAAAAGGAGGGATTAAATTGAAATACGGTTTCTTTGATGATAC	NarI is underlined
	2109BamDir	TTAAGGATCC**C**AGAATTTAAAAGGAGGGATTAAATTGAAATACGGTTTCTTTGATGATAC	BamHI is underlined
**Transcriptional links**	2107-E-RT-up	GGTACCGGACTCGGATTGT	
	2108-E-RT-up	GAAGGGCATCTAATGCCAGA	
	2108-S-RT-do	TCTGGCATTAGATGCCCTTC	
	2109-E-RT-up	TTATTAGCAATCCCGAACACG	
	2109-S-RT-do	TCTACGTAAACGTGCGTCCTT	
	2110-E-RT-up	GCCAGAATAGCAGCATTGGT	
	2110-S-RT-do	TGCTGTTGAGAGGACAGCAA	
	2111-E-RT-up	TGTATGAGGCTGCACGTGTT	
	2111-S-RT-do	CGTTCCATTCATTAAGGCTCA	
	2112-E-RT-up	GTGGGGATACATTGCTGACC	
	2112-S-RT-do	TGAGCTTGATGCAGAAGCAG	
	2113-E-RT-up	CGAAGTTGCAGAGAAGGTTG	
	2113-S-RT-do	ACCATCCATTCCAGGCATAC	
	2114-E-RT-up	CCGTTAATCGAAAATGCTGTC	
	2114-S-RT-do	TCGTTTGAGTACGAAAGGGATT	
	2115-E-RT-up	GGGGTACACCCAATAGCAAA	
	2115-S-RT-do	TTTGCTGTTACGAGCATTTGA	
	2116-E-RT-up	TCAGGAAGCCAGTGAAAAGG	
**qPCR**	Rpodir	AAACATAGTCAAGAAAGTAGAAAAG	
	Rporev	CTATACTAACAACCAGCCTTAAG	
	2109F	ATGAGTGCAGGCATGGTTTG	
	2109R	ACCGAGTTCTCAACTTCAACC	
	2110F	CACAGTTCAAGGCGTTCCAA	
	2110R	GCTCTGTACAACGCATCCAG	
	2111F	TAGCTGCAGGTTTGTTGTGG	
	2111R	CAACACGTGCAGCCTCATAC	

EXPERIMENT	NAME	SEQUENCE	FEATURES
**qPCR**	2112F	AGGAACCCTCTTTGACATGGA	
	2112R	CATTCTGGAAGGCAGCGATT	
	2113F	GCGGGGCATATGATTTCCTC	
	2113R	AGTCGTTTTCCATTACAACCAGA	
	2114F	CGGAACTCAGGTCAGTGGTA	
	2114R	CCTCGGATTCCAGTCCCTTT	
	2115F	TCCGTCCATGGGAAATGGAA	
	2115R	GCTGAATAGGCCGGATTCCT	
	2116F	ATTCAACCAGTCGGTCAGGA	
	2116R	ACCTACACCCGGTTCTCCTA	
