# Supplementary material for: A seven-gene cluster in Ruminiclostridium cellulolyticum is essential for signalization, uptake and catabolism of the degradation products of cellulose hydrolysis
Source: Biotechnol Biofuels. 2017 Oct 30;10:250. doi: 10.1186/s13068-017-0933-7 (PMC5663094; doi:10.1186/s13068-017-0933-7)
Supplement: Supplementary file 9 — Additional file 9. Analysis of membrane protein samples. 5 µg of the membrane fractions prepared from different strains and growth conditions are loaded on SDS-PAGE stained with Coomassie Blue before to be analyzed by Western blot. [file 13068_2017_933_MOESM9_ESM.pdf]

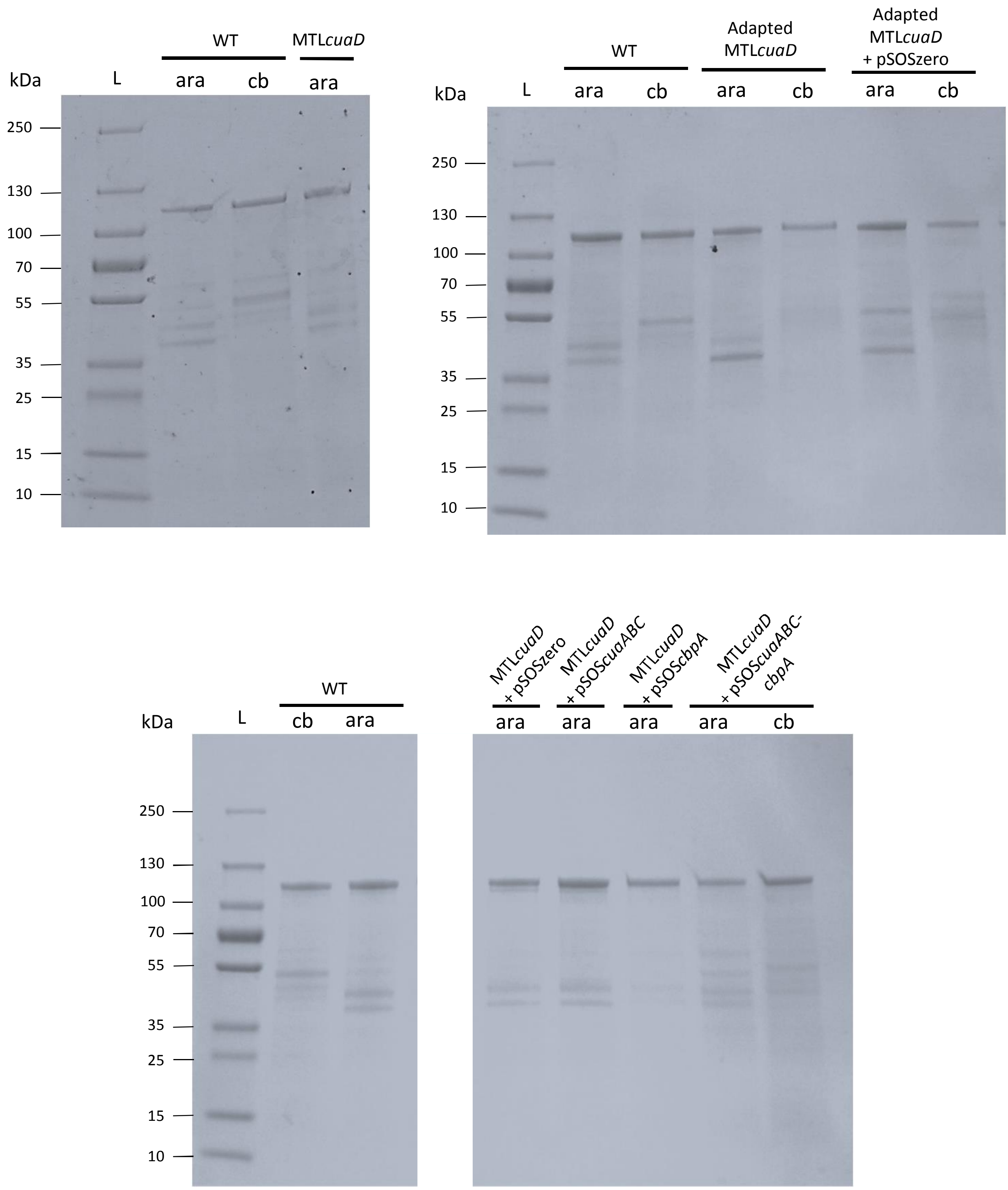

Additional file 9. Analysis of membrane protein samples. 5µg of the membrane fractions prepared from different strains and growth conditions are loaded on SDS-PAGE stained with Coomassie Blue before to be analyzed by Western blot.
